# “My [Search Strategies] Keep Missing You”: A Scoping Review to Map Child-to-Parent Violence in Childhood Aggression Literature

**DOI:** 10.3390/ijerph20054176

**Published:** 2023-02-26

**Authors:** Nikki Rutter

**Affiliations:** Department of Sociology, Durham University, Durham DH1 3HN, UK; nikki.rutter@durham.ac.uk

**Keywords:** child-to-parent violence, childhood aggression, scoping review, parent abuse, adolescent-to-parent violence

## Abstract

Child-to-parent violence is often referred to as one of the most ‘under-researched’ forms of family violence. However, it is closely associated with one of the most widely researched areas of research globally: childhood aggression. How child-instigated aggression can harm parents is widely referred to, but different framings, definitions, and conceptualisations are used which creates problems when attempting to identify the broader literature which may be relevant to child-to-parent violence researchers. Methods: Using the Preferred Reporting Items for Systematic Reviews and Meta-Analyses extension for Scoping Reviews, 55 papers were reviewed from EBSCO, PubMed, SCOPUS, and Web of Science to explore how location, field of the researcher, and terminology can impact how researchers conceptualise and frame this form of harm. Results: Three themes were identified (1) child-to-parent violence is a behavioural indicator of childhood distress or developmental needs, (2) children are ‘perpetrators’ of deviant behaviour, and (3) the parents are ‘victims’ of child-to-parent violence. Conclusions: Children and parents are both harmed by child-to-parent violence. It is important that future researchers and practitioners recognise the bi-directionality of the parent-child relationship, and not be complicit in hiding the harms caused by child-to-parent violence by subsuming it under the broader childhood aggression literature.

## 1. Introduction

‘Child-to-parent violence’ (CPV), as a form of child/adolescent-instigated harm, has proved difficult to understand and identify, not only because it does not fit our existing conceptualisations of family dynamics, but also due to the challenges associated with the lack of consensus regarding name, definition, description, or conceptual boundaries. Existing research has framed and conceptualised CPV as a form of domestic abuse [1]; utilised family-based conceptual approaches [2,3,4]; developmental approaches [5,6,7]; and socio-ecological models [8,9,10,11]. However, it maintains ‘fuzzy boundaries’ as whether it is related to power and control (domestic abuse), a developmental issue (behavioural), a mental health issue or personality type (pathologising), or a parenting problem (problematised) is unclear as current research is contradictory and predominantly exploratory [12].

Measuring the frequency of a phenomenon which has often been considered ‘hidden’ is difficult [13,14]. Studies have found a higher incidence in community samples, with physical CPV instigated by 4–22% of young people, and verbal/psychological CPV at 33–93% [11,15]; compared with policing samples, whereby CPV accounts for around 1–6% of reported cases of family violence [16]. In all samples which differentiate between the two, there appear to be higher rates of psychological CPV than physical CPV [17,18]. Regarding gender, there is debate as to whether CPV is predominantly instigated by sons or daughters, and this depends on the age of the child, and the definition of CPV used [11]. Definitions and measurements are further problematised in CPV research, as many recognised and validated measurements for CPV have been used outside the age range they were validated on [19]. Furthermore, how families themselves interpret and conceptualise this form of harm, means that CPV is often not framed as a form of family violence at all, but rather a form of childhood aggression, or a behavioural difficulty.

### 1.1. Violence and Aggression

Whilst CPV is often referred to as the most under-researched form of family violence, the broader field of childhood aggression is one of the most highly researched topics globally [20]. This paper is a scoping review of the existing literature on childhood aggression, in an attempt to identify where CPV may be discussed, but named differently, due to different conceptual approaches. The purpose of this paper is to identify where CPV exists in the existing literature, but is explored, understood and conceptualised differently depending on the underpinning societal, structural, and theoretical frameworks. If researchers, practitioners, and families are to benefit from the wealth of existing relevant research, it is important to be clear what relevant research exists which may be utilising different language, concepts, interpretations, or framing.

There are a number of examples regarding how CPV knowledge has changed over time. Several theories of children and childhood are related to child-instigated harms, including the Oedipal complex [21], early child development theories [22], and the most widely used, attachment theory, which is frequently used to demonstrate the importance of consistent responses of a parent to the needs of an infant, but originated from observations of children with ‘inappropriate’ behaviour [23]. The aim of this paper is to identify the existing literature which explores child-instigated harms within the home directed towards parents, and how it is conceptualised, named, and understood. The hypothesis is: the language used to define CPV will relate to the field of research in which it is used.

### 1.2. Operational Definition

Previous research has highlighted that CPV can be considered ‘at-odds’ with the broader childhood aggression literature due to a lack of identified harm in the latter [11]. Thus, I have opted to define CPV and its variations as ‘any form of physical, emotional, and psychological harm that may be instigated by a child towards their parent’. This includes coercive control, but more detailed inclusion and exclusion criteria will be presented later in this paper. Children refer to the UK legal definition of a ‘child’ being under the age of 18. Whilst this definition, unlike many other CPV definitions, does not require intent, it does require specific directionality whereby the harm is instigated by the child, and directed at the parent.

## 2. Materials and Methods

A scoping review is typically used for mapping the key concepts in a field, and to help with working definitions and conceptual boundaries, all of which are an issue in the field of CPV. It is not about measuring the quality of the literature, but about what is said about the topic of interest. Traditionally, systematic reviews require a team of reviewers to ensure consistency as well as reach, with a higher number of reviewers often reaching more papers and it has been recommended that multiple reviewers be involved in scoping reviews to increase their rigour and consistency [24,25]. However, where time and resources are limited, reviews with a single author can be effective, with the caveat that biases are acknowledged which may impact the number of papers included [25,26,27]. Due to the time and resource limitations of this paper, I have completed this review as a singular author, with an external reviewer selecting 15% of the papers to test the effectiveness of this approach.

### 2.1. Protocol

In this scoping review, I will follow a scoping review framework designed to examine “the extent, range and nature of research activity: this type of rapid review might not describe research findings in any detail but is a useful way of mapping fields of study where it is difficult to visualise the range of material that might be available” [28] (p. 21). This scoping review was reported according to the Preferred Reporting Items for Systematic Reviews and Meta-Analyses extension for Scoping Reviews (PRISMA-ScR) [29]. The initial search was implemented on 22 January 2022. This framework follows five stages, which I will now demonstrate.

#### 2.1.1. Stage One

For this stage, I identified the research question. As scoping reviews do not need a specific question but rather offer an opportunity to explore the boundaries and map the topic of interest I created the question ‘how do authors conceptualise the relevant phenomenon in their articles?’

#### 2.1.2. Stage Two

I utilised EBSO, PubMed, SCOPUS, and Web of Science core collection using the same search terms for each resource: children OR adolescents OR youth OR child OR teenager, AND violence OR aggression OR hostility OR violent OR anger OR aggressive behav*, AND externali* OR conduct disorder, child* AND Challenging behav*. The exact timeline of the search was 1961–2021 inclusive, as 1961 has been identified as a key point in which research began exploring circumstances of filicide, caused by children and adolescents [30,31].

#### 2.1.3. Stage Three

I outlined and tested inclusion criteria with five CPV-specific papers, and then followed inclusion and exclusion criteria to determine which articles should be included in the review. As the focus of the research was on children, the included papers must include children aged 0–18, rather than focus on harm instigated by adult children. The harm needed to be directional (towards a parental figure), within the home environment rather than in an institution or online, and there needed to be a description of the CPV or use of the term ‘explosive’. Therapeutic literature could be included only if there was also a conceptual description of CPV, and teacher ratings of CPV were not relevant.

The literature was excluded if it was not found within a peer-reviewed journal, if it was about uncovering the aetiology or predictors of occurrence, similarly studies on predictors of adult behaviour, prevention or intervention were not included. These included work which focused wholly on the relationship between victimisation and CPV. Parricide was considered a distinct offence [31], so was also not included. Self-harming or suicide studies which did not describe incidents of CPV were removed, as were experimental conditions, animal tests, and studies into substance misuse and its relationship to CPV.

**Figure 1:**
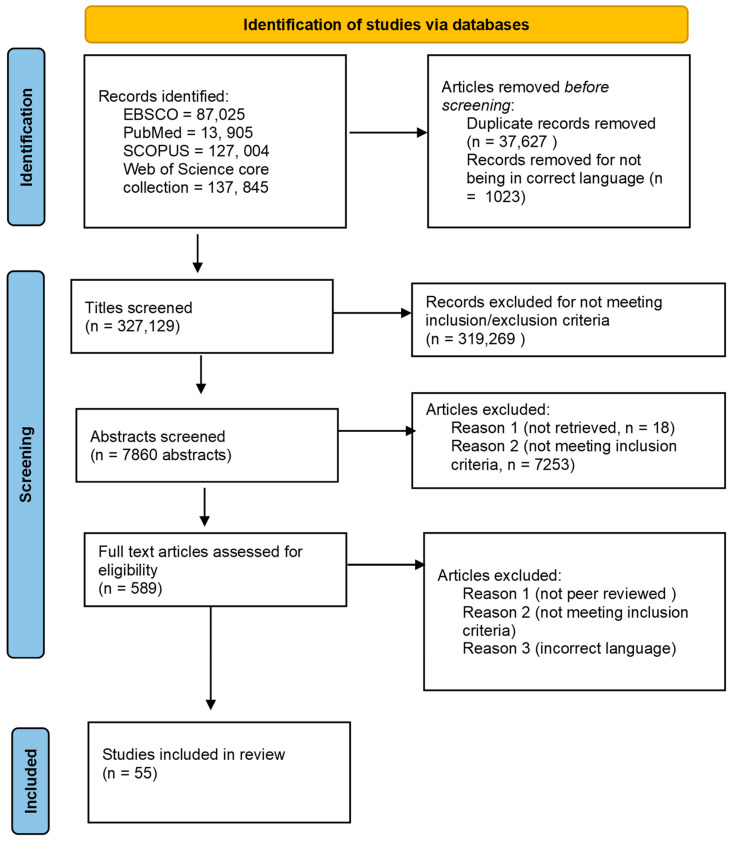
Adapted review procedure [32].

In this third stage, I reviewed titles, abstracts, and when relevant, the full-text papers. I included literature written in English and French, but articles in other languages were excluded as I could not translate them. For articles I could not access through my institution, but up-to-date contact details were available, I contacted the first author of the required paper to request a copy. Of the 20 authors contacted, two sent a copy of their article, with a cut-off date of 15 March 2022. Quantitative, qualitative, and mixed methods studies were included, provided they met the inclusion criteria. An external reviewer checked 15% of the papers to assess whether they would meet the inclusion/exclusion criteria with 55 articles included for the next stage. The review process is available at Figure 1.

#### 2.1.4. Stage Four

For this final stage, I mapped the final 55 articles onto a Microsoft Excel version 2212 sheet recording the following details accordingly:

- Author(s), year of publication, study location
- Study populations (parent population and child population)
- Aims of the study
- Main findings
- Methodology
- Language used to identify child-parent violence
- How the paper conceptualised the phenomenon

#### 2.1.5. Stage Five

This final stage was the analysis and writing up stage. Through the tabled literature included in the review, I conducted a frequency analysis, whereby I broke down the various fields, locations, data sources, and terminology used in an attempt to identify where and why certain terminology was preferred over others.

Following the analysis and write-up, a citation check of the identified literature was conducted for June 2021–February 2023 to capture any literature citing the relevant texts found in Table S1 which would have met inclusion criteria based upon the prior four stages, these 13 articles are available in Supplementary File S1 and have been integrated into the discussion section of this paper.

## 3. Results

How each article met the inclusion criteria is provided in Supplementary File S1, and includes the theoretical or conceptual underpinning. Where the theoretical underpinning has been identified as either domestic abuse or domestic violence, the former is related to the intent of harm, and the latter is related to the process of the harm. Of the 55 papers identified, 24 studies were qualitative [14,33,34,35,36,37,38,39,40,41,42,43,44,45,46,47,48,49,50,51,52,53,54,55]; 23 studies used quantitative methods [13,56,57,58,59,60,61,62,63,64,65,66,67,68,69,70,71,72,73,74,75,76,77], and eight papers used mixed methods [78,79,80,81,82,83,84,85]. When examining where each article was conducted, Most papers (23) were conducted in the USA [33,34,39,40,41,43,49,50,53,55,56,63,64,65,66,68,69,71,72,73,79,81,85]; 10 studies in the UK [13,14,35,36,38,46,52,82,83,84]; eight studies in Spain [37,57,59,60,61,67,75,76]; three in France [45,48,80]; three in Canada [51,74,78]; two in Australia [42,47]; one in New Zealand [54]; one in Belgium [44]; one in Egypt [58]; one in Mexico [70]; one in Chile [62]; and one in China [77].

Researchers came from a variety of fields, and whilst analysing this, I recorded the field of the first author when the team was interdisciplinary. The majority of research was conducted by a lead researcher in the broad field of psychology, with 16 papers [37,38,39,54,55,57,59,60,61,62,63,72,77,78,81,83]; the more specific fields of criminal psychology had two papers [14,46]; social psychology had three papers [67,75,76]; and clinical psychology had two [68,69]. Psychiatry was the second most common field in the review, with 10 papers [34,43,45,48,58,66,71,74,79,80]; and psychiatric nursing provided one paper [50]. Along similar lines, psychotherapy provided one paper [51]; as did psychosocial researchers [70].

From the review, 35/55 papers were based on the ‘psy’ field and its related underpinnings. Outside of the ‘psy’, there was one paper from the field of occupational therapy [47]; one from speech, language and hearing sciences [49], three from education [33,40,41], six from social work [35,52,64,73,84,85], one from youth justice [44], two from the law [36,42], four from criminology [13,53,56,82], and one from sociology [65].

There was a total of 135 different ways in which authors spoke about, or approached CPV (Table 1). From these, 58 of the different ways of referring to CPV gave specific information about who was instigating this form of harm, and recognised that it was directional (to parents, towards parents etc.); 53 of the different ways of referring to this phenomenon focused on the harm experienced by parents (mother abuse, parent battering etc.). However, most of the terminology used in the articles reviewed were very broad in their approach, using teminology such as ‘behaviour’, ‘aggression’, or ‘violence’, which may inform why this particular problem has remained ‘hidden’, as families request support for ‘aggression’, which could mean many things, rather than the specific issues experienced when living with CPV.

In Table 1, which papers, fields, and data sources to use for each terminology are identified. The majority of terms were only used in singular papers, rather than being adopted and repeated. The most commonly used term was ‘child-to-parent violence’, being used across 21 papers [13,14,37,46,47,52,54,56,57,60,61,62,63,65,67,70,73,75,76,82,84]. CPV was also used by the largest amount of fields and had the widest variety of data. It is likely that the inclusion criteria made this terminology most likely to appear, as terms needed to be directional and descriptive, however, it may also be due to the breadth of fields and data sources. Parent abuse is the second most commonly used term, with 10 references [39,46,47,58,60,61,72,79,81,82]. References to ‘abuse’ of parents throughout the review were most likely from psychology and specifically criminal psychology; there were also references from the law, criminology, occupational therapy, and social work, although the latter four fields had fewer articles overall than the former [14,36,37,39,40,46,47,52,58,60,61,72,74,78,79,81,82,84]. Twenty-six out of 55 papers directly referred to the phenomenon of interest as a form of violence from a child to a parent, meaning 29 papers did not. Highlighting the possibility of a large gap between the specific CPV literature, and the broader field of childhood aggression.

A large proportion of the literature consists of terms relating to ‘challenging behaviour’, behavioural problems, or similar [33,34,35,40,41,49,50,51,53,55,64,71,78,79,80,83,84,85], and there were several studies which focused on children with mental health [48,53,58,66,72,79] developmental [33,45,51,55], or neurodevelopmental needs [49,50,71,80,85]. Similarly, those who focused on preschool children used generalised approaches which included the aforementioned behavioural terminology but also utilised general terminology around ‘aggression’ [40,41] The data source with the broadest language base was in adoption and fostering, which covered challenging behaviour, distress, and had the highest number of terms related to coercion, and incidents understood to be controlling [34,35,84,85].

The results of this analysis could break the scoped literature into three key themes: (1) CPV as a behavioural indicator of childhood distress or developmental needs, (2) the lens of children/adolescents as ‘perpetrators’ of deviant behaviour, and CPV as a component of wider aggression or violent profiles, (3) the parents as ‘victims’ of CPV. In the discussion, I will explore how these three themes relate to the existing CPV literature and the potential challenges of integrating future work.

## 4. Discussion

As the scoped literature could be grouped into three separate themes, I present each of these themes in turn.

### 4.1. Indicating Childhood Distress or Developmental Needs

When exploring the challenges associated when parenting a child with developmental, mental health, or neurodevelopmental needs which are not met through traditional parenting strategies, many researchers refer to the parents as ‘carers’ rather than ‘parents’ and focus on the strain, burden, and stress experienced, rather than highlight the specificities of the harm [86]. This was also found in this review, as whilst the inclusion criteria required parents to be the ones experiencing ‘violence’, there were papers which referred to parents as “caregivers”, and positioned their child’s ‘challenging behaviour’ or ‘behavioural problems’ as a “burden”, rather than explore the bi-directionality of the parent-child relationship [33,50].

Children with challenging behaviour or harmful behavioural profiles appear to be more common in those with neurodevelopmental disabilities, and there has been some support for CPV to be considered within neurodevelopmental diagnostic assessments [33]. Many families seeking a neurodevelopmental diagnosis for their child described behaviours which would meet the criteria of CPV to be their main concern [33,87,88,89]. Thus, particularly in social work practice, it has been noted that patterns of behaviour consistent with CPV have been seen and understood, but interpreted as a form of ‘challenging behaviour’, and so the behavioural and relational patterns of CPV have remained hidden despite its prevalence [84,89].

The developmental component of CPV as an expression of childhood distress was present both within those children with neurodevelopmental conditions, and those younger children, who may not have developed the emotional regulation skills to manage their frustration or distress [40,41,52,68,69]. This was also demonstrated in the updating citation check, whereby some emotional or behavioural expressions which met the descriptive requirements for CPV, were harmful presentations of distress [88,90,91,92]. As to whether children are attempting to meet their needs in harmful ways has the potential to create an escalation of harmful behaviour over time if those children have needs continue to go unmet [12,34,35,78].

‘Emotional and behavioural difficulties and similar terminology were found in the scoping review [35,55,71,78,79,85], however, it is possible that this form of language is conflating issues, as extant literature provides evidence that behavioural indicators are more significant than emotional indicators when looking at how emotional and behavioural difficulties are related to CPV [61,63]. This provides some challenges around understanding risks associated with expressions of mental health difficulties, as whilst externalising behaviours may represent CPV [64,68,69,78,85], mental health difficulties alone will not necessarily mean CPV, despite the number of scoped articles focusing on mental health [48,53,58,66,72,79].

### 4.2. Indicating Deviance

This theme explored how children and young people instigating CPV demonstrated a pattern indicative of a wider aggressive or violent profile, whereby it was conceived that something was inherently ‘wrong’ or harmful about the way in which the child or young person engaged with the world, rather than approaching CPV as a structural or systemic problem.

Some researchers conceived that CPV could be evidence that the child or young person had a criminal personality profile [63,74,76], or psychopathological traits which promoted such harm [36,66,83]. In the fields of research, it would be expected that psychiatric papers would promote this approach to interpreting CPV. However, the number of psychiatric articles that presented with this framing [34,79], were matched by social work [73,84], despite social work practice being underpinned by anti-oppressive principles. Social work papers, particularly those with a recruitment strategy focusing upon adoption and the perspectives of parents, were the most likely to frame this form of harm as controlling, and coercive [64,84]. The also sat alongside criminologists in the broader research field which often conceptualised CPV as a form of domestic abuse [13,42,44,54,56,57,62,67,70,73,82].

In framing CPV as a form of domestic abuse, it is likely that the specific concerns and risk factors which are distinct from adult-perpetrated intimate partner violence could be ignored, as CPV is subsumed under the wider field of research and practice [60,77]. This is of particular concern when children straddle the identity of both victims of harm, and the cause of the harm [47,93]. It should not be ignored that children are children first, thus when their CPV is harming their relationships, this is bi-directional and escalatory, thus children should also be provided with support alongside their families in navigating this harm [47,93,94].

### 4.3. Prioritising Parents as Victims

Differing to viewing children as ‘perpetrators’, is the conceptualisation of CPV whereby parents are considered to be ‘victims’ of CPV. In much previous literature, there was an exploration of how poor mental health in children can impact their aggression and violent behaviour, thus taking a more generalised not directional approach, meaning those harmed by the aggression (parents) were invisible [57]. Similarly, due to the ‘less significant levels of harm caused by CPV in comparison to intimate partner violence, the longer term impacts of the ham were ignored [72,79]. However, this approach has changed, with increasing awareness of both the victimisation and stigmatisation of parental experiences [14,37].

As to which fields recognise CPV as a form of victimising parents is contextual, and relates to both the field of research and the participant groups [91,95]. Where it is clear that the topic of investigation was explicitly CPV, any reference to aggression, coercion, or harm was clearly related to CPV, and thus there was more opportunity to explore the experiences of parents. However, when the research explored a broader field, such as challenging behaviour, then who is impacted by the harm was less clear because the directionality of the behaviour is unclear, and one argument as to why there has been an increasing awareness of this form of harm, reflects other forms of family violence whereby there is increasing recognition that family violence is a social problem as much as a private one [46].

Where parental experiences of victimisation were most clear, was in literature whereby mothers had experienced domestic abuse historically, and their child was either being weaponised by an abusive father or was repeating the harm [96]. Related to this, children who had experienced intimate partner violence in their parent’s relationship, and then began instigating CPV themselves have been considered to be both compounding how mothers are victimised by violence, whilst also being weaponised and victimised themselves, as children instigating such harm are also experiencing harm [97].

## 5. Conclusions

Children who instigate CPV hold the joint identity of victim and perpetrator, and the significance of harm caused to young people through their use of harm should not be conflated with other forms of victimisation, as it is a unique form of harm which requires specific intervention [47,97]. Rather than viewing children as ‘perpetrators’, and parents as ‘victims’, there appears to be some benefit in recognizing the bi-directionality of parent-caregiver-child relationships.

Many CPV researchers describe the phenomenon as “under-researched”, including myself previously [52], which I now consider to be an incomplete perspective, as demonstrated by this review. There is significant overlap between all of the conceptual framings presented, with significant overlaps as all conceptualisations gave an example whereby child-instigated harms were considered a symptom of a wider development issue, or one component of a wider pattern of harmful behaviours. Similarly, harms instigated by children and experienced by parents are often understood as an expression of distress. Very few articles framed it in a way which fits with existing forms of violence and abuse, thus demonstrating that as the broader field of family violence was recognised as a social problem, not a private one, it brought with it CPV without considering the often ‘hidden’ nature of child-rearing/parenting [98,99,100].

To conflate the CPV experiences of families under the umbrella of family violence risks losing the nuanced essence of family experiences of child-instigated harms. The differences in approaches and language appear to be related to the positionality and training of the researcher or practitioner and ignores the processes that families engage with when navigating, facilitating, or avoiding the harms instigated by their children. Thus, it is understandable that many researchers and practitioners consider CPV as a problem facing adolescents and their families, whereas younger children are considered ‘challenging’ or ‘problematic’ rather than harmful. This is reflected in the extant research as, despite childhood aggression being one of the most commonly researched forms of human behaviour, it is usually related to early childhood development, framed as a ‘pathological’ or development issue, or framed as ‘challenging behaviour’.

### 5.1. Limitations

Whilst this scoping review has provided a significant contribution when attempting to understand how CPV research may be conceptualised and understood across different fields and research groups, it does not provide the systematic body of knowledge that could address how to explore this field in a robust and inclusive way that is provided by systematic reviews. Furthermore, the vast number of studies that were identified in the initial scoping stages does evidence that many studies could be referring to CPV or challenging behaviour, but lack a detailed definition or description of the phenomenon of interest. Thus, many papers may not have been included which provide even broader interpretations of CPV.

As with many scoping reviews, the inclusion criteria are broad enough to capture the boundaries of many fields, but not specific enough to provide specific recommendations for how we move forward. Furthermore, scoping reviews do not require registration and validation through PROSPERO systematic review systems. Indeed, the challenges relating to naming, definitions, and interpretation remain. Furthermore, there are no recommendations for supporting parents with children who would otherwise be considered experiencing a ‘caregiver burden’ rather than recognising their direct experiences of harm. Nevertheless, the integration of such a wide scope of literature under three themes provides the opportunity to expand our knowledge of this phenomenon, and how it can impact families in their day-to-day interactions.

This review did not include literature which focused on prediction, risk, or aetiology of harm, thus it is unclear whether those factors could have fit within the themes presented here, or been included under new themes. There have been other scoping studies connected to exploring these features, but a meta-analysis is worth considering in the future. There is a significant body of literature which has developed in Spain, which meant many studies could not be included as they could not be translated, this is a significant limitation at this point, due to the wealth of Spanish studies on this topic written in Spanish.

### 5.2. Recommendations

Within practice and research, consideration should be given to the directionality of harmful or distressing behaviours expressed by children and adolescents, whilst also being mindful that language, concepts, and interpretations may not adequately represent the experience of participants or service users. Parents may be using the terminology of ‘challenging behaviour’, but mean significant harm. As was evidenced in this work, descriptions of the behaviour may assist in the understanding of what families are referring to when they use different concepts, as they may not be translated appropriately across to practitioners when engaging in help-seeking behaviours.

## Figures and Tables

**Table 1:** Language used.

Name	Articles	Field	Data Source
Badly behaved	[34]	Psychiatry	Fostered child in a therapeutic clinic
Behavioural manifestations	[79]	Psychiatry	Clinic case examples
Coping or survival behaviours	[85]	Social Work	Adopted children
Challenging behaviour	[33,35,40,41,49,51,55,83,84]	Education; Social work; Speech, language and hearing sciences; Psychology	Disabled children and their parents; Parents of adopted children; Parents of preschool children; Community sample of adolescents; Families seeking support for their children’s behaviour
Tyrannical behaviour	[80]	Psychiatry	Parents of children with oppositional defiance
Disruptive behaviours	[34,50,83]	Psychiatry; Psychiatric nursing; Psychology	Fostered child in therapeutic clinic; Adolescents in treatment for behavioural issues; Community sample of 101 boys and their parents
Tantrum-hit sequences	[68]	Clinical psychology	Infants under 2
Psychiatric difficulties	[78]	Psychology	Foster parents
Externalising behaviours	[64,68,69,85]	Social work; Clinical psychology	Families with adopted children; Infants under 2
Emotional and behavioural difficulties	[35,78,79,85]	Social work; Psychology; Psychiatry	Parents of adopted children; Foster parents; Child outpatients in a mental health clinic
Emotional or behavioural challenges	[55,78]	Psychology	Families seeking support for their children’s behaviour; Foster parents
Problem behaviours	[49,64]	Social work; Speech, language and hearing sciences	Families with adopted children; Parents of disabled children
Problematic behaviours	[50]	Psychiatric nursing	Families of adolescents with disruptive behaviour disorders
Conduct problems	[83]	Psychology	Community sample of 101 boys and their parents
Behavioural problems	[53,71,85]	Psychiatry; Social work; Criminology	Parents of children with neurodevelopmental disabilities; Adopted children; Families who self-reported living with a child with mental illness
Behavioural issues	[53]	Criminology	Families who self-reported living with a child with mental illness
Deviant behaviours	[44]	Youth Justice	Youth at a public protection centre
Rage	[45,48,85]	Psychiatry; Social work	9-year-old boy with disability; Records of children hospitalised with mental health issues; Parents of adopted children
Anger	[69]	Clinical psychology	Infants under 2
Acts of resistance	[51]	Psychotherapy	Mothers accessing support for their child’s challenging behaviours
Adolescent conflict	[64]	Social work	Parents of adopted adolescents
Defiance	[68]	Clinical psychology	Infants under 2
Altercations	[50]	Psychiatric nursing	Families of adolescents with disruptive behaviour disorders
Aggression	[40,45,51,60,67,72,74,81]	Education; Psychology, Psychotherapy; Psychiatry; Social work; Social psychology	Parents of pre-schoolers; Parents of adopted children; Mothers of 15-year-old adolescents; Mothers in a domestic abuse refuge; Mothers accessing support for their child’s challenging behaviours; 9-year-old boy with disability; Children in mental health outpatient settings; Youth justice settings; Adolescents in secondary school
Presenting with aggression	[43]	Psychiatry	11-year-old autistic boy
Children who are aggressive	[84]	Social Work	Parents of adopted children
Psychological aggression	[57]	Psychology	School sample
Verbal aggression	[41,49,57,72,84,85]	Psychology; Education; Speech, language and hearing sciences; Social work	School sample; Parents of pre-schoolers; Mothers of children with developmental disability (Fragile X); Parents of adopted children; Child mental health outpatients
Physical and verbal aggression	[39]	Psychology	Mothers in a domestic abuse refuge
Verbal, psychological, and emotional aggression	[46]	Criminal psychology	Practitioners
Aggression from children toward parents	[74]	Psychiatry	Mothers of 15-year-old children
Verbal, physical, psychological, emotional or financial harm to parents	[54]	Psychology	Families with lived experience
Verbal and physical aggression toward mothers	[50,74]	Psychiatry; Psychiatric nursing	Families of adolescents with disruptive behaviour disorders; Mothers of 15-year-old children
Physical aggression toward parents and caregivers	[72]	Psychology	Child mental health outpatients
Physical altercations with adults	[50]	Psychiatric nursing	Families of adolescents with disruptive behaviour disorders
Physical aggression	[41,49,51,68,69,84,85]	Education; Clinical psychology; Speech, language and hearing sciences; Psychotherapy; Social work	Parents of children under 2; Mothers of children with developmental disability (Fragile X); Mothers seeking support for their child’s challenging behaviour; Parents of adopted children
Aggressive children	[34,66]	Psychiatry; Criminology	Children in a psychiatric service; 10 individuals from four families who self-reported living with a child with mental illness
Aggressive behaviour	[43,50,53,66,74,80,84]	Psychiatry; Psychiatric nursing; Social work; Criminology	Parents of children with behavioural ‘disorders’; Mothers of 15-year-old children; Parents of adopted children; Autistic children; Children in a psychiatric service; Families who self-reported living with a child with mental illness
Aggressive outbursts	[43]	Psychiatry	11-year-old autistic boy
Violent outbursts	[53]	Criminology	Families who self-reported living with a child with mental illness
Outbreaks of violence	[48]	Psychiatry	Hospital records of children hospitalised with mental health issues
Violent episodes	[48]	Psychiatry	Hospital records of children hospitalised with mental health issues
Explosive, irritable or angry	[71]	Psychiatry	Parents of children with neurodevelopmental disabilities
Property destruction and abuse	[41]	Education	Parents of pre-schoolers
Destructive	[71]	Psychiatry	Parents of children with neurodevelopmental disabilities
Explosive, oppositional, and aggressive	[71]	Psychiatry	Parents of children with neurodevelopmental disabilities
Explosive, oppositional, and aggressive behaviour	[71]	Psychiatry	Parents of children with neurodevelopmental disabilities
Aggressive behaviour toward parents	[48]	Psychiatry	Hospital records of children hospitalised with mental health issues
Aggression towards parents	[59,70,73,77,81]	Psychology; Psychosocial; Social work	Mothers in a domestic abuse refuge; Community sample of adolescents; Youth arrested for domestic battery against their mothers
Aggression towards others	[40]	Education	Parents of pre-schoolers
Aggression towards mothers	[60]	Psychology	Policing data
Parent-directed aggression	[39,81]	Psychology	Mothers in a domestic abuse refuge
Parent-directed physical aggression	[72]	Psychology	Child mental health outpatients
Violence directed against parents	[44]	Youth Justice	Youth at a public protection centre
Youth violence directed toward significant others	[65]	Sociology	High school youth and youth referred to a youth justice centre
Perpetrates violent acts against their parents	[42]	Law	Practitioner focus groups
Crimes against a caregiver	[63]	Psychology	Children and adolescents referred to the Juvenile Court Assessment Centre
Youth offenders who use violence against their parents	[76]	Social psychology	Youth involved in youth justice due to violence against parents
Violence by adolescents towards their parents	[61]	Psychology	Policing data and community sample of youth
Violence against the parent	[48]	Psychiatry	Hospital records of children hospitalised with mental health issues
Violence against one’s own parents	[48]	Psychiatry	Hospital records of children hospitalised with mental health issues
Violence against parents	[38,65,76]	Psychology; Social psychology; Sociology	Adolescent; High school students; Youth involved in youth justice due to violence against parents
Violence towards parents;	[14]	Criminal psychology	Parents experiencing violence
Violence directed at parents	[70]	Psychology	Youth in schools
Violence towards a parent	[63]	Psychology	Children and adolescents referred to the Juvenile Court Assessment Centre
Violence towards their parents	[13]	Criminology	Police data
Filial violence	[46]	Criminal psychology	Practitioners
Violence	[66]	Psychiatry	Children in psychiatric services
Sons’ violence	[60]	Psychology	Police data
Violent child	[53]	Criminology	Families who self-reported living with a child with mental illness
Violence from children	[13]	Criminology	Police data
Violence from children to parents	[62]	Psychology	Adolescents in secondary school
Violence of adolescents toward their parents	[48]	Psychiatry	Medical records of child mental health inpatients
Violent behaviour towards parents	[63]	Sociology	High school youth and youth referred to a youth justice centre
Violent behaviours against parents	[60]	Psychology	Police data
violent behaviour directed by juveniles against members of their own family	[65]	Sociology	High school youth and youth referred to a youth justice centre
Violent behaviour	[53,66]	Psychiatry; Criminology	Children in a psychiatric service; Families who self-reported living with a child with mental illness
Violence and destructiveness	[38]	Psychology	Adolescents
Adolescent-to-parent violence	[13,36,46,52,75,82,84]	Law; Criminology; Criminal psychology; Social psychology; Social work	Case examples; Police data; Practitioners; Mothers of pre-adolescent children; Parents of adopted children; Community sample
Adolescents who are violent towards their family members	[42]	Law	Practitioners
Adolescents who are violent towards their parents	[47]	Occupational therapy	Parents and practitioners
Adolescent violence to parents	[47]	Occupational therapy	Parents and practitioners
Adolescent violence in the home	[47]	Occupational therapy	Parents and practitioners
Youth who are violent in the family	[73]	Social Work	Youth arrested for domestic battery against their parent
Adolescent-to-parent physical aggression	[77]	Psychology	Adolescents in school
Physical and psychological aggressions perpetrated against the mother	[62]	Psychology	Adolescents in school
Adolescent-to-mother psychological aggression	[77]	Psychology	Adolescents in school
Child-to-mother violence	[47,59,73]	Psychology; Occupational therapy; Social work	Adolescents from a community sample; Parents and practitioners; Youth arrested for domestic battery against their mother
Child-to-father violence	[59]	Psychology	Adolescents from a community sample
Child-to-parent violence	[13,14,37,46,47,52,54,56,57,60,61,62,63,65,67,70,73,75,76,82,84]	Social work; Sociology; Criminology; Psychology; Social psychology; Criminal psychology; Occupational therapy	Police data; Parent and professional focus groups; Children and adolescent school samples; Parents of children and adolescents instigating this harm; Parents of adopted children; Children and adolescents referred to the Juvenile Court Assessment Centre; Practitioners; Youth arrested for domestic battery against their mothers; Community sample
Child-to-parent aggression	[77]	Psychology	Adolescents in school
Child-to-parent aggression and violence	[14]	Criminal psychology	Parents of children and adolescents instigating this harm
Child-to-parent violence or abuse	[14]	Criminal psychology	Parents of children and adolescents instigating this harm
Child-to-parent abuse	[58]	Psychiatry	Adolescent mental health outpatients
Parents who are abused by their adolescent children	[74]	Psychiatry	Mothers of 15-year-old children
Parent abuse	[39,46,47,58,60,61,72,79,81,82]	Psychiatry; Psychology; Criminal psychology; Criminology; Occupational therapy	Clinic case examples; Child and adolescent mental health outpatients; Mothers in a domestic abuse refuge; Parents; Practitioners; Policing data
Mother abuse	[47,84]	Occupational therapy; Social work	Parents and practitioners; Parents of adopted children
Adolescent abuse towards parents	[47]	Occupational therapy	Parents and practitioners
Adolescents who assaulted their parents	[37]	Psychology	Parent and professional focus groups
Juveniles who assault their parents	[61]	Psychology	Policing data and community sample of youth
Parental maltreatment	[74]	Psychiatry	Mothers of 15-year-old children
Abused parents	[79]	Psychiatry	Clinic case examples
Abuse of parents	[37]	Psychology	Focus group of parents and practitioners
Abuse	[14,36,42,54,57,60,78]	Law; Psychology; Criminal psychology	Case examples; Foster parents; School sample; Professional focus groups; Parents of children and adolescents instigating this harm; Policing data
Abusing	[42]	Law	Practitioners
Verbal and Physical Abuse Toward Mothers	[74]	Psychiatry	Mothers of 15-year-old children
Physical abuse	[53,79,80]	Psychiatry; Criminology	Clinic case examples; Families who self-reported living with a child with mental illness
Verbal abuse	[53,79]	Psychiatry; Criminology	Clinic case examples; Families who self-reported living with a child with mental illness
Abusive children	[79]	Psychiatry	Clinic case examples
Abusive behaviour	[53]	Criminology	Families who self-reported living with a child with mental illness
Abusive behaviour towards mothers	[74]	Psychiatry	Mothers of 15-year-old children
Abusive actions perpetrated by children and adolescents towards their parents	[54]	Psychology	Families with lived experience
Violent abusers	[42]	Law	Practitioners
Youth who perpetrate violence against a parent	[73]	Social Work	Youth arrested for domestic battery against their mother
Violence perpetrated by children against their parents	[48]	Psychiatry	Medical records of child mental health inpatients
Violence and abuse perpetrated against parents	[82]	Criminology	Policing data
Adolescent family violence	[42,47]	Law; Occupational therapy	Parents; Practitioners
Family Violence	[44,59]	Youth Justice; Psychology	Youth at a public protection centre; Adolescents
Intrafamily violence	[48,59]	Psychology; Psychiatry	Adolescents; Medical records of child mental health inpatients
Domestic violence incident	[56]	Criminology	Policing data
Physical violence	[75]	Social psychology	Community sample of adolescents
Psychological violence	[75]	Social psychology	Community sample of adolescents
Coercive behaviour	[64]	Social work	Parents of adopted adolescents
Violent, controlling and coercive behaviours	[84]	Social Work	Parents of adopted children
Controlling behaviours	[84]	Social Work	Parents of adopted children
Psychological control	[80]	Psychiatry	Parents of children with oppositional defiance
Violence	[53,84]	Social Work; Criminology	Parents of adopted children; Families who self-reported living with a child with mental illness
Harm	[46]	Criminal psychology	Practitioners
Harmful act by an adolescent against a parent	[73]	Social Work	Youth arrested for domestic battery against their mother
Non-homicidal physical attacks	[79]	Psychiatry	Clinic examples
Physically assaulting parents	[57]	Psychology	School sample
Parent battering	[48]	Psychiatry	Medical records of child mental health inpatients
Children who batter their parents	[74]	Psychiatry	Mothers of 15-year-old children
Battered parent syndrome	[48,80,82]	Psychiatry; Criminology	Parents of children with oppositional defiance; Medical records of child mental health inpatients; Police data

## Data Availability

Data can be accessed by contacting the corresponding author.

## Supplementary Materials

The following supporting information can be downloaded at: https://www.mdpi.com/article/10.3390/ijerph20054176/s1.

## References

[1] Molla-Esparza C., Aroca-Montolío C. (2018). Menores que maltratan a sus progenitores: Definición integral y su ciclo de violencia. Anu. Psicol. Juríd..

[2] Almagro-García P., Cutillas-Poveda M.J., Sánchez-Villegas S., Sola-Ocetta M. (2019). Fuerza exterior, debilidad interior. Ejes fundamentales de la violencia filio-parental. Rev. Sobre Infanc. Adolesc..

[3] Hunter C., Nixon J., Parr S. (2010). Mother abuse: A matter of youth justice, child welfare or domestic violence?. J. Law Soc..

[4] Micucci J.A. (1995). Adolescents who assault their parents: A family systems approach to treatment. Psychother. Theory Res. Pract. Train..

[5] Connor D.F., Newcorn J.H., Saylor K.E., Amann B.H., Scahill L., Robb A.S., Jensen P.S., Vitiello B., Findling R.L., Buitelaar J.K. (2019). Maladaptive aggression: With a focus on impulsive aggression in children and adolescents. J. Child Adolesc. Psychopharmacol..

[6] Holt A., Shon P.C. (2018). Exploring fatal and non-fatal violence against parents: Challenging the orthodoxy of abused adolescent perpetrators. Int. J. Offender Ther. Comp. Criminol..

[7] Weymouth B.B., Buehler C., Zhou N., Henson R.A. (2016). A meta-analysis of parent–adolescent conflict: Disagreement, hostility, and youth maladjustment. J. Fam. Theory Rev..

[8] Bronfenbrenner U. (1979). The Ecology of Human Development: Experiments by Nature and Design.

[9] Arias-Rivera S., García M.V.H. (2020). Theoretical framework and explanatory factors for child or adolescent-to-parent violence and abuse. A scoping review. An. Psicol..

[10] Cottrell B., Monk P. (2004). Adolescent-to-parent abuse: A qualitative overview of common themes. J. Fam. Issues.

[11] Simmons M., McEwan T.E., Purcell R., Ogloff J.R. (2018). Sixty years of child-to-parent abuse research: What we know and where to go. Aggress. Violent Behav..

[12] Rutter N. (2022). “It’s Like Living in a House with Constant Tremors, and Every So Often, There’s an Earthquake” A Glaserian Grounded Theory Study into Harm to Parents, Caused by the Explosive and Controlling Impulses of Their Pre-Adolescent Children. Ph.D. Thesis.

[13] Condry R., Miles C. (2014). Adolescent to parent violence: Framing and mapping a hidden problem. Criminol. Crim. Justice.

[14] Holt A. (2011). ‘The terrorist in my home’: Teenagers’ violence towards parents–constructions of parent experiences in public online message boards. Child Fam. Soc. Work.

[15] McCloud E.J. (2017). Adolescent-to-Parent Violence and Abuse (APVA): An Investigation into Prevalence, Associations and Predictors in a Community Sample. Unpublished Ph.D. Thesis.

[16] Moulds L., Day A., Mayshak R., Mildred H., Miller P. (2019). Adolescent violence towards parents—Prevalence and characteristics using Australian Police Data. Aust. N. Z. J. Criminol..

[17] Peek C.W., Fischer J.L., Kidwell J.S. (1985). Teenage violence toward parents: A neglected dimension of family violence. J. Marriage Fam..

[18] Sampedro R., Calvete E., Gámez-Guadix M., Orue I. Child-to-Parent Aggression in adolescents: Prevalence and reasons. Proceedings of the 16th European Conference on Developmental Psychology, International Proceedings Division.

[19] Simmons M.L., McEwan T.E., Purcell R., Huynh M. (2019). The Abusive Behaviour by Children-Indices (ABC-I): A measure to discriminate between normative and abusive child behaviour. J. Fam. Violence.

[20] Liu J., Lewis G., Evans L. (2013). Understanding aggressive behaviour across the lifespan. J. Psychiatr. Ment. Health Nurs..

[21] Freud S. (1924). The passing of the Oedipus complex. Int. J. Psycho-Anal..

[22] Freud A. (1949). Notes on aggression. Bull. Menn. Clin..

[23] Bowlby J. (1953). Some pathological processes set in train by early mother-child separation. J. Ment. Sci..

[24] Levac D., Colquhoun H., O’Brien K.K. (2010). Scoping studies: Advancing the methodology. Implement. Sci..

[25] Waffenschmidt S., Knelangen M., Sieben W., Bühn S., Pieper D. (2019). Single screening versus conventional double screening for study selection in systematic reviews: A methodological systematic review. BMC Med. Res. Methodol..

[26] Hazel N. (2005). Holidays for children and families in need: An exploration of the research and policy context for social tourism in the UK. Child. Soc..

[27] Ibabe I. (2020). A systematic review of youth-to-parent aggression: Conceptualization, typologies, and instruments. Front. Psychol..

[28] Arksey H., O’Malley L. (2005). Scoping studies: Towards a methodological framework. Int. J. Soc. Res. Methodol..

[29] Tricco A.C., Lillie E., Zarin W., O’Brien K.K., Colquhoun H., Levac D., Moher D., Peters M.D., Horsley T., Weeks L. (2018). PRISMA extension for scoping reviews (PRISMA-ScR): Checklist and explanation. Ann. Intern. Med..

[30] Easson W.M., Steinhilber R.M. (1961). Murderous aggression by children and adolescents. Arch. Gen. Psychiatry.

[31] Walsh J.A., Krienert J.L. (2009). A decade of child-instigated family violence: Comparative analysis of child—Parent violence and parricide examining offender, victim, and event characteristics in a national sample of reported incidents, 1995–2005. J. Interpers. Violence.

[32] Page M.J., McKenzie J.E., Bossuyt P.M., Boutron I., Hoffmann T.C., Mulrow C.D., Shamseer L., Tetzlaff J.M., Akl E.A., Brennan S.E. (2021). The PRISMA 2020 statement: An updated guideline for reporting systematic reviews. BMJ.

[33] Adams N.B., McGuire S.N., Meadan H., Martin M.R., Terol A.K., Haidar B., Fanta A.S. (2021). Impact of Challenging Behaviour on Marginalised and Minoritised Caregivers of Children with Disabilities. Top. Early Child. Spec. Educ..

[34] Ament A. (1972). The boy who did not cry. Child Welf..

[35] Bardsley K. (2017). Adopting children with high therapeutic needs: Staying committed over the long haul. Adopt. Foster..

[36] Bettinson V., Quinlan C. (2020). De-criminalising adolescent to parent violence under s 76 Serious Crime Act 2015 (c. 9). J. Crim. Law.

[37] Calvete E., Orue I., Bertino L., Gonzalez Z., Montes Y., Padilla P., Pereira R. (2014). Child-to-parent violence in adolescents: The perspectives of the parents, children, and professionals in a sample of Spanish focus group participants. J. Fam. Violence.

[38] Campbell R. (1967). Violence in adolescence. J. Anal. Psychol..

[39] Desir M.P., Karatekin C. (2018). Parent-and sibling-directed aggression in children of domestic violence victims. Violence Vict..

[40] Doubet S.L., Ostrosky M.M. (2015). The impact of challenging behaviour on families: I don’t know what to do. Top. Early Child. Spec. Educ..

[41] Doubet S.L., Ostrosky M.M. (2016). Parents’ experiences when seeking assistance for their children with challenging behaviours. Top. Early Child. Spec. Educ..

[42] Douglas H., Walsh T. (2018). Adolescent family violence: What is the role for legal responses?. Syd. Law Rev..

[43] Frank G.K. (2013). An 11-year-old boy with Asperger’s disorder presenting with aggression. Am. J. Psychiatry.

[44] Helin D., Chevalier V., Born M. (2004). Ces adolescents qui agressent leur mère!. Neuropsychiatr. Enfance Adolesc..

[45] Holman T. (2009). Le «crying boy». Imagin. Inconsc..

[46] Holt A., Lewis S. (2021). Constituting child-to-parent violence: Lessons from England and Wales. Br. J. Criminol..

[47] Kehoe M., Ott N., Hopkins L. (2021). Responding to Adolescent Violence in the Home–A Community Mental Health Approach. Aust. N. Z. J. Fam. Ther..

[48] Laurent A., Derry A. (1999). Violence of French adolescents toward their parents: Characteristics and contexts. J. Adolesc. Health.

[49] Muller K., Brady N.C., Warren S.F., Fleming K.K. (2019). Mothers’ perspectives on challenging behaviours in their children with fragile X syndrome. J. Intellect. Dev. Disabil..

[50] Oruche U.M., Draucker C.B., Al-Khattab H., Cravens H.A., Lowry B., Lindsey L.M. (2015). The challenges for primary caregivers of adolescents with disruptive behaviour disorders. J. Fam. Nurs..

[51] Robson J., Kuczynski L. (2018). Deconstructing noncompliance: Parental experiences of children’s challenging behaviours in a clinical sample. Int. J. Qual. Stud. Health Well Being.

[52] Rutter N. (2021). “I’m meant to be his comfort blanket, not a punching bag”–Ethnomimesis as an exploration of maternal child to parent violence in pre-adolescents. Qual. Soc. Work.

[53] Sporer K. (2019). Aggressive children with mental illness: A conceptual model of family-level outcomes. J. Interpers. Violence.

[54] Williams M., Tuffin K., Niland P. (2017). “It’s like he just goes off, BOOM!”: Mothers and grandmothers make sense of child-to-parent violence. Child Fam. Soc. Work.

[55] Worcester J.A., Nesman T.M., Mendez L.M.R., Keller H.R. (2008). Giving voice to parents of young children with challenging behavior. Except. Child..

[56] Armstrong G.S., Muftic L.R., Bouffard L.A. (2021). Factors Influencing Law Enforcement Responses to Child to Parent Violence. J. Interpers. Violence.

[57] Cortina H., Martín A.M. (2020). The behavioural specificity of child or adolescent-to-parent violence and abuse.Explores frequency of different forms. An. Psicol..

[58] Fawzi M.H., Fawzi M.M., Fouad A.A. (2013). Parent abuse by adolescents with first-episode psychosis in Egypt. J. Adolesc. Health.

[59] Hoyo-Bilbao D., Orue I., Gámez-Guadix M., Calvete E. (2020). Multivariate models of child-to-mother violence and child-to-father violence among adolescents. Eur. J. Psychol. Appl. Leg. Context.

[60] Ibabe I., Jaureguizar J. (2010). Child-to-parent violence: Profile of abusive adolescents and their families. J. Crim. Justice.

[61] Ibabe I., Arnoso A., Elgorriaga E. (2014). The clinical profile of adolescent offenders of child or adolescent-to-parent violence and abuse. Procedia Soc. Behav. Sci..

[62] Jiménez-García P., Pérez B., Contreras L., Cano-Lozano M.C. (2020). Analysing child or adolescent-to-parent violence and abuse in Chilean adolescents: Prevalence and reasons. Curr. Psychol..

[63] Kennedy T.D., Edmonds W.A., Dann K.T., Burnett K.F. (2010). The clinical and adaptive features of young offenders with histories of child-parent violence. J. Fam. Violence.

[64] Koh B.D., Rueter M.A. (2011). Contributions of parent–adolescent negative emotionality, adolescent conflict, and adoption status to adolescent externalising behaviours. J. Clin. Child Adolesc. Psychol..

[65] Kratcoski P.C. (1985). Youth violence directed toward significant others. J. Adolesc..

[66] Lewis D.O., Shanok S.S., Grant M., Ritvo E. (1983). Homicidally aggressive young children: Neuropsychiatric and experiential correlates. Am. J. Psychiatry.

[67] López-Martínez P., Montero-Montero D., Moreno-Ruiz D., Martínez-Ferrer B. (2019). The Role of Parental Communication and Emotional Intelligence in Child-to-Parent Violence. Behav. Sci..

[68] Lorber M.F., Del Vecchio T., Slep A.M.S. (2014). Infant externalising behaviour as a self-organising construct. Dev. Psychol..

[69] Lorber M.F., Del Vecchio T., Slep A.M.S. (2015). The emergence and evolution of infant externalising behaviour. Dev. Psychopathol..

[70] Martínez-Ferrer B., Romero-Abrio A., León-Moreno C., Villarreal-González M.E., Musitu-Ferrer D. (2020). Suicidal ideation, psychological distress and child-to-parent violence: A gender analysis. Front. Psychol..

[71] Mayes S.D., Calhoun S.L., Aggarwal R., Baker C., Mathapati S., Anderson R., Petersen C. (2012). Explosive, oppositional, and aggressive behaviour in children with autism compared to other clinical disorders and typical children. Res. Autism Spectr. Disord..

[72] Nock M.K., Kazdin A.E. (2002). Parent-directed physical aggression by clinic-referred youths. J. Clin. Child Adolesc. Psychol..

[73] Nowakowski-Sims E., Rowe A. (2017). The relationship between childhood adversity, attachment, and internalising behaviours in a diversion program for child-to-mother violence. Child Abus. Negl..

[74] Pagani L., Larocque D., Vitaro F., Tremblay R.E. (2003). Verbal and physical abuse toward mothers: The role of family configuration, environment, and coping strategies. J. Youth Adolesc..

[75] Seijo D., Vázquez M.J., Gallego R., Gancedo Y., Novo M. (2020). Adolescent-to-parent violence: Psychological and family adjustment. Front. Psychol..

[76] Vecina M.L., Chacón J.C., Piñuela R. (2021). Child-to-parent violence and dating violence through the moral foundations theory: Same or different moral roots?. Front. Psychol..

[77] Zhang L., Cai C., Wang Z., Tao M., Liu X., Craig W. (2019). Adolescent-to-Mother psychological aggression: The role of father violence and maternal parenting style. Child Abus. Negl..

[78] Brown J.D., Bednar L.M. (2006). Foster parent perceptions of placement breakdown. Child. Youth Serv. Rev..

[79] Charles A.V. (1986). Physically abused parents. J. Fam. Violence.

[80] Delaunay E., Purper-Ouakil D., Mouren M.C. (2008). Troubles oppositionnels de l’enfant et tyrannie intrafamiliale: Vers l’individualisation de types cliniques. Ann. Med. Psychol..

[81] Desir M.P., Karatekin C. (2018). Parental reactions to parent-and sibling-directed aggression within a domestic violence context. Clin. Child Psychol. Psychiatry.

[82] Miles C., Condry R. (2016). Adolescent to parent violence: The police response to parents reporting violence from their children. Polic. Soc..

[83] Roberts R., McCrory E., Joffe H., De Lima N., Viding E. (2018). Living with conduct problem youth: Family functioning and parental perceptions of their child. Eur. Child Adolesc. Psychiatry.

[84] Selwyn J., Meakings S. (2016). Adolescent-to-parent violence in adoptive families. Br. J. Soc. Work.

[85] Smith S.L., Howard J.A., Monroe A.D. (2000). Issues underlying behaviour problems in at-risk adopted children. Child. Youth Serv. Rev..

[86] Meltzer H., Ford T., Goodman R., Vostanis P. (2011). The burden of caring for children with emotional or conduct disorders. Int. J. Fam. Med..

[87] Boxall H., Sabol B. (2021). Adolescent family violence: Findings from a group-based analysis. J. Fam. Violence.

[88] Holt A., Birchall J. (2022). ‘Their Mum Messed Up and Gran Can’t Afford to’: Violence towards Grandparent Kinship Carers and the Implications for Social Work. Br. J. Soc. Work.

[89] O’Toole S.E., Tsermentseli S., Papastergiou A., Monks C.P. (2022). A Qualitative Exploration of Practitioners’ Understanding of and Response to Child-to-Parent Aggression. J. Interpers. Violence.

[90] Gervais C., DeCarlo-Slobodnik D., Romano E. (2022). Parental Perspectives on Upholding Children’s Rights in the Context of Aggression toward Family/Caregivers in Childhood & Adolescence (AFCCA) in Canada. Can. J. Child. Rights Rev. Can. Droits Enfants.

[91] Lyttle E., McCafferty P., Taylor B.J. (2021). Experiences of adoption disruption: Parents’ perspectives. Child Care Pract..

[92] Papamichail A., Bates E.A. (2022). “I want my mum to know that I am a good guy…”: A thematic analysis of the accounts of adolescents who exhibit child-to-parent violence in the United Kingdom. J. Interpers. Violence.

[93] Laflamme E., Matte-Gagné C., Baribeau-Lambert A. (2022). Paternal mind-mindedness and infant-toddler social-emotional problems. Infant Behav. Dev..

[94] Toole-Anstey C., Townsend M.L., Keevers L. (2022). ‘He’s out of control, I’m out of control, it’s just–I’ve got to do something’: A narrative inquiry of child to parent violence. Child Adolesc. Soc. Work J..

[95] Condry R., Miles C. (2021). Children who perpetrate family violence are still children: Understanding and responding to adolescent to parent violence. Young People Using Family Violence: International Perspectives on Research, Responses and Reforms.

[96] Abbaspour Z., Vasel G., Khojastehmehr R. (2021). Investigating the Lived Experiences of Abused Mothers: A Phenomenological Study. J. Qual. Res. Health Sci..

[97] Meyer S., Reeves E., Fitz-Gibbon K. (2021). The intergenerational transmission of family violence: Mothers’ perceptions of children’s experiences and use of violence in the home. Child Fam. Soc. Work.

[98] Cuervo K. (2021). A deeper understanding of child to parent violence (CPV): Personal traits, family context, and parenting. Int. J. Offender Ther. Comp. Criminol..

[99] Navas-Martínez M.J., Cano-Lozano M.C. (2022). Differential profile of specialist aggressor versus generalist aggressor in child-to-parent violence. Int. J. Environ. Res. Public Health.

[100] Baeza P.A.I., Fiscella J.M.G. (2021). Adolescents who are violent toward their parents: An approach to the situation in Chile. J. Interpers. Violence.

