# Supplementary material for: “My [Search Strategies] Keep Missing You”: A Scoping Review to Map Child-to-Parent Violence in Childhood Aggression Literature
Source: Int J Environ Res Public Health. 2023 Feb 26;20(5):4176. doi: 10.3390/ijerph20054176 (PMC10001475; doi:10.3390/ijerph20054176)
Supplement: Supplementary file 1 [file ijerph-20-04176-s001.zip › Supplementary File S1.pdf]

May 2021 – February 2023 [13,14,33–85]

Article	Relevant articles	Method	Theme
[13,82]	[88]	Described and compared different adolescent family violence ‘offenders’ in Australia.	Indicating deviance
[13,85]	[89]	Interviews with kinship carers and practitioners where there is child-to-parent violence	Childhood distress
[57,60,68]	[90]	Focus groups with 25 practitioners working in the field of CPV	Indicating deviance
[14,38,47,74,85]	[91]	Canadian research utilising interviews with 18 caregivers recruited through adoption services of children instigating CPV	Childhood distress
[14,73,85]	[93]	Narrative inquiry with 11 mothers experiencing CPV	Parents as victims
[36]	[94]	Adoption disruption in Northern Ireland	Parents as victims
[38,62,74,85]	[95]	UK-based accounts from young people instigating CPV	Childhood distress
[38,59,61,75]	[96]	Interviews with mothers experiencing CPV in Iran	Parents as victims
[43,83]	[97]	Interviews with Australian mothers who were experiencing CPV after IPV	Parents as victims/childhood distress
[49,64,73,75,85]	[98]	Spanish study with youth offenders of CPV	Indicating deviance
[60,68,71,77]	[99]	Questionnaire with 1559 Spanish CPV offenders	Indicating deviance
[61,62,73,75,80]	[100]	An analysis of CPV within Chile	Indicating deviance
[69,70]	[101]	Observations of parent-child interactions and parental questionnaires of their toddlers	Child distress
