# Supplementary material for: “My [Search Strategies] Keep Missing You”: A Scoping Review to Map Child-to-Parent Violence in Childhood Aggression Literature
Source: Int J Environ Res Public Health. 2023 Feb 26;20(5):4176. doi: 10.3390/ijerph20054176 (PMC10001475; doi:10.3390/ijerph20054176)
Supplement: Supplementary file 1 [file ijerph-20-04176-s001.zip › Supplementary Table S1.pdf]

Supplementary Table S1. Meeting criteria

Paper	Description	Conceptual or theoretical underpinning
[13]	"Assaults upon parents by under 18-year-olds".	Domestic abuse
[14]	"All of the accounts posted referred to physical violence, which was often described as co-occurring with verbal abuse. Thus, parents referred to being bitten, kicked, battered, thumped and punched – in the throat, head and stomach."	Domestic violence
[34]	"People who would see the behavior are going to think, 'Oh, look at him. He's violent. What's wrong? He's hitting his parents.'"	Bronfenbrenner ecological model
[35]	"Allan had responded to discipline by temper tantrums and by kicking the previous foster parents".	Therapeutic approach
[36]	"They spoke of losing friends, of being physically hurt [by their adopted child]".	Therapeutic approach
[37]	"Young people use a variety of physical and psychological behaviours to hurt their parent and often with the goal of getting what they want".	Domestic abuse
[38]	"When they are distressed, with whom do they get upset? Whom do they mistreat? The person who is there, whom they love and whom they can trust. In the end, he lashes out at me because he knows he can".	Psychosocial approach
[39]	"An account of an adolescent boy in whom the turmoils and stresses of adolescence were exaggerated and augmented until his aggressive feelings boiled over into violence against his parents and his home".	Therapeutic approach
[40]	"Physical and verbal aggression and emotional blackmail were the most common forms of aggression against caregivers".	Domestic violence
[41]	"He hits me, kicks me and his sister. Tantrums. Terrible. I walk away. Screaming, kicking, bounces his head off the wall, the floor, whatever, for attention. He does not hurt himself."	Psychoanalytical
[42]	"Brandt has hit a hole in his wall. He'll throw toys, break pictures in his room. He has hurt his sister and me. He hits me, kicks me, and his sister".	Ecological
[43]	"An older child, generally postpubescent, perpetrates violent acts upon their parents, siblings or other members of their household"	Domestic abuse
[44]	"His parents' chief complaint at admission was Alan's physically aggressive behavior at home, in particular toward his mother"	Therapeutic
[45]	"Les conséquences de cette nouvelle forme de violence sont généralement bénignes au niveau physique (ecchymoses, griffures), mais plus importantes au niveau psychologique. (The consequences of this new form of violence are generally benign at the physical level (bruises, scratches),but more important at the psychological level)".	Ecological
[46]	"Les affrontements quotidiens entre Jimmy et sa mère la rendent anxieuse, déprimée et épuisée. Pendant ces conflits, il la frappe souvent, lui donne des coups de pied, ou la mord. (The daily clashes between Jimmy and his mother make her anxious, depressed and exhausted. During these conflicts, he hits her. Often kicks her, or bites her)."	Therapeutic approach

[47]	"One family support worker was unable to find support services for a family where two 15- and 20-year-old girls 'were severely beating Mum on a daily basis' and felt that the issue 'wasn't taken seriously because it was daughters against Mum"	Domestic violence
[48]	"'My son has me in a choke hold. When I looked up and saw my gorgeous 8-year-old daughter holding a golf club I knew I had to do something"	Systems approach
[49]	"Coerces his parents to do his homework and to send excuse notes for his numerous absences from school... Whenever his parents attempt to set up limits, he insults, strikes, or bites them".	Ecological
[50]	"I tell him no and then I get slapped. If I'm trying to take him to the grocery store or Walmart he'll blow an absolute fit. He'll slap; he'll hit; he'll kick if he can"	Psychopathological
[51]	"The primary caregivers described being pushed, slapped, and choked by the adolescents. One adolescent hit a caregiver "in the mouth" and another threw a phone book at his caregiver. A number of caregivers described how the adolescents brandished objects or weapons during these altercations."	Double ABCX Model of Family Stress and Adaptation
[52]	"Mothers also reported aggression towards themselves or other family members. For example, one mother reported that her son attacked her, 'He has, he's been very aggressive at times. So that's the one when he has come at me'".	Grounded Theory
[53]	"If a man was punching me in the chest and screaming in my face they'd tell me to go, that he'll kill me, that I've got to put myself first… But, no, 'cos it's [my son] I've got to work something out".	Phenomenology
[54]	"His first psychiatric hospitalization occurred before he turned 13 after he aggressively assaulted his mother".	Symbolic interactionism and family systems theory
[55]	"Parents are threatened, intimidated and controlled by their children"	Phenomenology
[56]	"Lenny is unpredictable and he's destructive not only to our own family unit, but to people around him."	Developmental, phenomenology
[57]	"A child allegedly assaulted his or her parent".	Domestic abuse
[58]	"Behaviors aimed at controlling and/or causing physical, psychological, emotional or economic harm to parents"	Domestic abuse
[59]	"The cultural taboo against striking or abusing parents".	Psychosocial
[60]	"147 offenders were recruited from eight Spanish specialized closed or semi-closed institutions for adolescents who had shown aggression toward their parents"	Domestic abuse
[61]	"abuse toward one's parents frequently begins with verbally abusive episodes and progresses over time in frequency and intensity, escalating to emotional and physical abuse"	Domestic abuse
[62]	"Juveniles who assault their parents"	Psychosocial
[63]	"The study of the reasons provided by adolescents for attacking their parents is key to understanding the nature of this phenomenon".	Psychosocial
[64]	"Juvenile offenders who committed violent crimes [any behavior that involves the direct use of physical aggression] against a parent"	Domestic violence

[65]	"Adolescent conflict was defined as hostile, angry, and coercive adolescent behavior directed toward each parent"	Transactional
[66]	"Youth violence against parent [(pushed, shoved, slapped, beaten with fist or an object, threatened with gun, knife, or other weapon]"	Social learning; stress theory; subculture
[67]	"Of the 21 homicidally aggressive children, 9 had attacked siblings, 5 had attacked mothers".	Ecological/therapeutic
[68]	"It is common for CPV to start with the economic form before progressing to psychological levels and reaching physical violence, to the point that all three types are eventually exercised simultaneously"	Psychosocial
[69]	"physical aggression, defiance, activity level, and distress to limitations… propose that patterns among these individual externalizing behaviors form in 'real time,' often in interactions with caregivers".	Systems theory
[70]	"if the infant is capable of identifying a parent as the source of her or his frustration, she or he may angrily (i.e., be distressed by limitations) react against her (i.e., aggress) and attempt to pursue his or her own desires despite parental resistance (i.e., defiance)".	Developmental
[71]	"repeated behaviors of physical, psychological, or economic violence directed at parents"	Socio-ecological; general strain theory
[72]	"Mothers rated their children… measuring explosive, oppositional, and aggressive behavior"	Psychopathological
[73]	"children have physically harmed their parents"	Ecological
[74]	"A sample of 80 youth who have been arrested for domestic battery against a mother".	Trauma-informed; attachment theory
[75]	"The child, feeling a great sense of entitlement and a grandiose sense of self, threatens or physically attacks the parent in response to the constraint imposed by the parent"	Ecological
[76]	"I slapped or punched my father/mother".	Ecological
[77]	"The participants were 136 young and violent individuals who had been sentenced in court for various violent acts either against their parents".	Moral foundation theory
[78]	"Many parents who have been attacked psychologically by their children do not get the support or professional help they need".	Ecological
[79]	"The presence of violence by foster children, and its effects on [foster carers] in the home".	Systems approach
[80]	"a 9-year-old girl, who was hospitalized after an attempt to stab her mother with a kitchen knife".	Psychosocial
[81]	"Repeated physical and psychological abuse and familial dysfunction characterized by an inversion of familial authority".	Psychopathology
[82]	"Caregivers who reported that their children had aggressed against them"	Domestic violence
[83]	"the threat or use of violence and/or damage to property from 13 to 19 year olds towards their parents"	Domestic violence
[84]	"'…sometimes it's like living with a volcano waiting for it to explode…'"	Ecological
[85]	"Include coercive control, domination and intimidation".	Trauma-informed, developmental approach
[86]	"Upon referral he is full of rage and physically abusive to his adoptive mother."	Psychosocial
